# Advances in Molecular Medicine: Unravelling Disease Complexity and Pioneering Precision Healthcare

**DOI:** 10.3390/ijms241814168

**Published:** 2023-09-16

**Authors:** Stephen A. Bustin, Kurt A. Jellinger

**Affiliations:** 1 Medical Technology Research Centre, Anglia Ruskin University, Chelmsford CM1 1SQ, UK; 2 Institute of Clinical Neurobiology, Alberichgasse 5/13, A1150 Vienna, Austria

The escalating impacts of the climate crisis, zoonotic spill-over, and antibiotic resistance have positioned molecular medicine at the forefront of pioneering translational research. With the rapid advances in modern medicine, the constantly evolving technologies of molecular pathology, diagnostics, and therapeutics are driving the transformation towards precision medicine. By exploring the molecular intricacies of diseases, these inter-related disciplines offer novel insights into disease origins and progressions and promise the identification of more effective therapeutic targets. Consequently, the growing integration of these fields has the potential to revolutionise healthcare by accelerating the advent of personalised, targeted, and more effective treatments. This combination also plays a pivotal role in improving the ability to make informed choices for managing infectious and metabolic diseases. Molecular pathology drives the development and validation of biological diagnostic assays, facilitating the identification of disease-specific biomarkers or pathogens. Molecular diagnostics, in turn, helps guide treatment decisions, influences the most appropriate molecular therapeutics for an individual patient’s unique profile, and is an essential guardian of public health. As molecular therapeutics and treatment responses are recorded, valuable insights are fed into molecular pathobiology and diagnostics research, refining biomarker discovery and therapeutic strategies.

Molecular pathology focuses on the study and interpretation of molecular and genetic changes in tissues and cells to aid in the diagnosis, pathogenesis, prognosis, and treatment of diseases. By analysing genetic mutations, gene expression patterns, epigenetic modifications, and protein alterations, molecular pathologists aim to understand the underlying mechanisms driving disease development and progression. Hence, molecular pathology differs from molecular diagnostics in its broader scope and application. While molecular diagnostic mainly focus on detecting specific biomarkers, molecular pathologists work closely with clinicians, oncologists, and other healthcare professionals to correlate specific molecular alterations with histopathological findings, biomarkers, clinical data, and patient outcomes. This comprehensive interaction aims to provide a solid foundation for the accurate diagnosis, prognostication, and prediction of treatment responses, thereby significantly improving patient outcomes.

Molecular pathology plays a critical role in bridging the gap between basic research and clinical applications. The identification of disease-specific biomarkers and genetic alterations provides insight into pathogenic mechanisms and enables the development of targeted therapies and personalised treatment regimens. The ability to classify diseases and stratify patients precisely, based on their molecular profiles, facilitates the implementation of tailored therapeutic approaches, thereby maximising treatment efficacy and minimising adverse effects. Additionally, molecular pathology supports advancements in research and drug development by elucidating molecular targets and pathways that can be exploited for novel therapeutic interventions.

Molecular pathology is witnessing rapid advances in technology and automation, enabling higher-throughput analysis and improved accuracy. Integrating data from various ‘-omics’ technologies will yield a more comprehensive understanding of disease pathogenesis. Furthermore, artificial intelligence and machine learning algorithms are anticipated to revolutionise disease classification and prognosis, empowering clinicians with more precise and personalised treatment recommendations.

Molecular diagnostics is crucial in guiding targeted therapies, predicting treatment responses, and improving overall patient care through its precise and rapid testing capabilities. It has two main applications:

- The first involves applying molecular techniques to detect and analyse genetic and molecular biomarkers in patient samples, thus defining the era of personalised medicine. By identifying disease-specific biomarkers, molecular diagnostics plays a pivotal role in early detection, accurate diagnosis, prognosis, and treatment monitoring and has started to transform the landscape of disease detection and management.
- The second involves diagnosing and screening infectious diseases, where rapid and sensitive molecular tests have proven particularly vital in the COVID-19 pandemic. The ability to identify SARS-CoV-2 as its causative agent quickly and accurately was instrumental in developing tests, targeted therapeutics, and vaccines. More generally, traditional methods for infectious disease diagnosis are time-consuming and may lack sensitivity, leading to delays in treatment and potential disease transmission. Molecular diagnostics offer the potential for the rapid and accurate detection of infectious agents, allowing for timely intervention, infection control, and improved patient outcomes.
- The third involves applying molecular biomarkers in the diagnosis and screening for diseases in the hepatic and gastrointestinal systems, including neoplasms, often hiding behind non-specific symptoms, where the application of biomarkers enables a rapid, non-invasive, and accurate diagnosis, as well as continuous monitoring of therapeutic interventions and patient outcomes.

Molecular biomarkers encompass a wide array of molecules, including nucleic acids, proteins, lipids, and metabolites, all of which are potential indicators of disease status or therapeutic responses. Techniques like the polymerase chain reaction (PCR) allow for the rapid and sensitive detection of target genetic sequences and quantification of gene expression signatures, as well as enabling the identification of pathogens with high sensitivity and specificity. Next-generation sequencing technologies have further advanced the ability to comprehensively analyse the genetic makeup of pathogens, providing crucial insights into their virulence, resistance mechanisms, and epidemiological patterns. Enzyme-linked immunosorbent assays (ELISAs) and mass spectrometry enable the quantification and profiling of additional biomarkers in patient samples, providing valuable information about disease progression and treatment efficacy.

The significance of molecular diagnostics lies in its ability to detect diseases at their earliest stages, enabling timely intervention and improved patient outcomes. Early detection can prevent disease progression, reduce treatment complexities, and lead to more successful therapeutic interventions. Moreover, molecular diagnostics offers a powerful tool for monitoring treatment response and detecting potential relapse, guiding clinicians to adjust treatment regimens accordingly. It also empowers genetic counsellors to provide personalised risk assessments, thus facilitating informed decision-making for individuals with inherited genetic conditions. 

Molecular diagnostic testing also enables the detection of drug-resistant strains of infectious agents, aiding in selecting appropriate antimicrobial therapies. At the same time, identifying genetic markers associated with drug resistance allows clinicians to tailor treatments to optimise patient outcomes and minimise the development of further resistance.

The future of molecular diagnostics is poised for remarkable advancements driven by emerging technologies and innovative methodologies. Liquid biopsies, which involve analysing circulating tumour DNA, RNA, and other biomolecules, hold immense promise for non-invasive early cancer detection and monitoring. Furthermore, point-of-care molecular diagnostic devices are likely to become more accessible and widely adopted, particularly in resource-limited settings, thereby democratising precision medicine. As the field continues to evolve, integrating digital health platforms and data-sharing initiatives will streamline the exchange of molecular diagnostic information, fostering collaboration and enabling evidence-based decision making.

In the context of genetic analysis, integrating genomic data with electronic health records and machine learning algorithms holds great promise for precision medicine. This integration will enable the identification of personalised treatment options based on individual genetic profiles, leading to improved therapeutic outcomes and reduced adverse effects.

In infectious diseases, the continuous development of novel molecular techniques will contribute to faster and more widespread pathogen detection, facilitating the early intervention and containment of outbreaks. Moreover, ongoing research into host–pathogen interactions and immune responses will advance our understanding of infectious disease pathogenesis, leading to identifying new therapeutic targets and developing effective antiviral and antimicrobial agents.

Molecular therapeutics describes a wide range of therapeutic approaches that utilise our understanding of molecular pathways, genetics, and biomarkers to develop targeted treatments for various diseases, including personalised medicine and targeting some infectious diseases. This can involve targeted therapies, such as monoclonal antibodies or small molecule inhibitors, that specifically act on key molecules or pathways involved in disease processes. In the context of infectious diseases, it can be aimed at disrupting the entry and replication of pathogens or allowing the development of vaccines targeting specific antigens, as for SARS-CoV-2. Unlike traditional therapies that often result in non-specific systemic effects, molecular therapeutics focuses on targeting the root cause of the disease, preserving healthy tissues and minimising adverse reactions. The significance of molecular therapeutics lies in its potential to usher in a new era of customised medicine. By tailoring treatments to individual patients based on their unique molecular profile, molecular therapeutics maximise treatment efficacy while minimising the risk of treatment resistance and side effects. This approach has shown tremendous promise in managing various diseases, particularly cancer. By specifically targeting molecular aberrations in cancer cells, targeted therapies have shown remarkable success in prolonging survival, improving quality of life, and achieving remission in previously untreatable cases. Beyond cancer, molecular therapeutics holds potential for managing autoimmune disorders, rare genetic diseases, and infectious diseases, heralding a new era of precision medicine with tailored treatments for individual patients.

The future of molecular therapeutics promises exciting developments in precision drug design and delivery. Advances in pharmacogenomics will enable the identification of patient-specific drug responses, avoiding treatments that may prove ineffective or harmful. Combinatorial therapies involving a combination of targeted agents, immunotherapies, and conventional treatments are expected to emerge as powerful treatment regimens with synergistic effects. Moreover, gene editing technologies have the potential to correct genetic defects and treat genetic disorders at the molecular level, opening up new therapeutic avenues. Finally, the success of RNA vaccines against diseases like COVID-19 has demonstrated the power of molecular therapeutics in rapidly responding to infectious disease outbreaks.

This Special Issue can only touch on a small section of the vast and diverse landscape that this field encompasses. Nonetheless, the issue explores a broad spectrum, from mechanosensing through fibroids, cancer, and inflammatory conditions to myocardial infarction. This Special Issue also takes us beyond traditional medical territories, delving into the realm of mindfulness-based interventions. The exciting explorations of how mindfulness can influence molecular biomarkers open new doors for integrative approaches to managing mental health and fostering overall well-being. The 16 papers presented in this Special Issue represent a remarkable collection of contributions highlighting the richness and complexity of molecular pathology, diagnostics, and therapeutics.

The first review deals with infectious diseases, particularly mycobacterium tuberculosis, the cause of tuberculosis (TB), a highly contagious airborne disease affecting billions worldwide [1]. One of the concerning aspects of TB is its ability to remain latent in the body for years, during which many individuals become asymptomatic carriers. Approximately one-quarter of the world’s population, about 2 billion people, are latently infected with M. tuberculosis, and while most latent carriers never develop active TB, the risk of TB reactivation is 5–10% over a lifetime. However, it is significantly higher in immunosuppressed people, particularly if they are co-infected with HIV. Current front-line treatments for drug-sensitive strains involve a 6-month protocol with four drugs, but drug-resistant strains have emerged, necessitating longer and more toxic treatments. In the last decade, only three new drugs were approved for TB treatment, and their novel modes of action provide potential options for patients with drug-resistant strains. Research efforts are ongoing to identify promising preclinical and clinical anti-TB drug candidates that inhibit new protein targets in M. tuberculosis. Research into the molecular mechanisms underlying M. tuberculosis’ pathogenesis and the factors regulating its growth and virulence is paving the way for novel strategies in TB treatment.

The second review deals with solid organ transplantation (SOT) [2], a life-saving treatment for end-stage organ failure, but with demand for transplants exceeding organ availability. One major challenge is the lack of non-invasive biomarkers to monitor transplanted organs. Recently, extracellular vesicles (EVs) have shown promise as biomarkers for various diseases. In SOT, EVs facilitate communications between donor and recipient cells, carrying valuable information about graft function. They could be used for preoperative assessment, postoperative monitoring, and diagnosing rejection, infection, or other issues. The review summarises the recent evidence on using EVs as biomarkers in SOT and discusses their potential clinical applications, especially their use as biomarkers for long-term graft survival and improved patient outcomes.

Tourkochristou and colleagues [3] highlight the importance of VDR signalling in non-alcoholic fatty liver disease (NAFLD) and its potential as a therapeutic target. NAFLD is a chronic liver condition with growing global incidence. It is a complex disease with diverse phenotypes, and pharmacological treatment options are still limited. The vitamin D/vitamin D receptor (VDR) axis is closely linked to NAFLD development and progression, and VDR gene polymorphisms may influence NAFLD severity by affecting adipose tissue activity, fibrosis, and hepatocellular carcinoma (HCC) development. Vitamin D binds to hepatic VDR, regulating gene expression associated with inflammation and fibrosis. VDR activity has protective and detrimental effects on hepatic steatosis, a characteristic feature of NAFLD. Understanding the genetic and molecular background of VDR in NAFLD could lead to new therapeutic targets, including developing VDR agonists, which have shown promising results. 

The next review discusses mechanosensing, which describes the ability of a cell to sense and respond to mechanical cues of its microenvironment [4]. It plays an essential role in tissue function, with tissues like muscles, bones, tendons, and cartilage requiring mechanical loading for proper function. Conversely, mechanical unloading leads to pathological tissue remodelling and dysfunctions. At the cellular level, mechanical loading and unloading regulate various pathways, including those involving reactive oxygen species (ROS). The Nuclear Factor-E2-related factor 2/Antioxidant response element (Nrf2/ARE) system plays a crucial role in maintaining the redox balance in cells and tissues. The dysregulations of this system have been linked to liver, neurodegenerative, and cancer diseases. This review explores the role of the Nrf2 system in mechanosensitive tissues, highlighting its importance for tissue functionality and emphasising the need for further research in the context of redox biology.

Yang and Al-Hendy’s review summarises the recent progress in ascertaining the biological functions and regulatory mechanisms in Uterine fibroids (UFs) [5]. These are benign tumours of the myometrium affecting many women worldwide and, although non-cancerous, can cause significant health issues and reproductive problems. The excessive accumulation of the extracellular matrix (ECM) is a key characteristic of UFs, and understanding the regulation of ECM production and signalling may offer new treatment strategies. The molecular mechanisms underlying UFs are poorly understood, and risk factors such as race, age, and genetic factors are also known to contribute to UF development. Despite the significant health and financial burden UFs impose, there are currently no specific therapeutics due to the complexity and variability of the condition. More research is required to improve our understanding and develop targeted treatments for UFs. 

The following three reviews discuss how new therapeutic strategies for cancer treatment, particularly targeted therapies, have improved patient survival. One of the main challenges remains the lack of understanding of the mechanisms of resistance to conventional therapies, with primary and secondary drug resistance remaining a substantial problem for many patients. Acquired resistance is often determined using the overexpression of efflux pumps, and the review by Labbozzetta [6] discusses drug resistance in patients with acute myeloid leukaemia (AML), where a subgroup of patients becomes refractory to all available therapies. This is due to overexpressed efflux pumps, notably P-glycoprotein (P-gp). The conventional inhibitors of P-gp have shown toxicity issues due to the inhibition of the physiological functionality of P-gp in healthy tissues, leading to a recent focus on natural molecules with low toxicity. These substances can target the overexpressed pump in neoplastic cells without affecting normal cells. The review describes four natural substances—phytol, lupeol, curcumin, and heptacosane—that can target P-gp via various mechanisms, including inhibiting its expression at the transcriptional level or acting as substrate inhibitors. Additionally, Labbozzetta discusses whether they may have a place in the clinic as adjuvants in chemotherapy regimens, especially in the niche category of patients with relapsing and resistant AML.

The second of the three articles reviews the development of new treatment options for HCC [7], the most common primary liver cancer and a leading cause of cancer-related deaths globally. Categorising HCC patients has always been problematic, given the frequent presence of cirrhosis and HCC. This leads to competing risk factors for death and can make treatment decisions difficult, as the need to preserve normal liver function while simultaneously eliminating the cancer is a frequent paradigm treating physicians face. While systemic therapies have shown some benefits, their efficacy remains limited, prompting the focus on locoregional therapies to address this complex disease. Most locoregional HCC trials have been conducted in patients without extra-hepatic disease, making locoregional therapies a primary choice for those with limited disease burden. On the other hand, trials evaluating systemic therapies have predominantly involved patients with extra-hepatic disease, leading to systemic therapy being the primary strategy for this population. However, an important subset of patients with locally advanced HCC overlaps between both therapies. These patients, often with macrovascular invasion, pose unique challenges and opportunities for combined treatment approaches involving locoregional and immunologically active therapies. The review focuses on two treatment strategies, emphasising the potential of combining locoregional therapies with checkpoint inhibitors to improve outcomes in locally advanced HCC: immunotherapies and locoregional therapies. The review discusses the available data on the immunomodulatory effects of locoregional therapies and the clinical data on outcomes when combining the two strategies. 

The third review provides an update on photodynamic therapy (PDT) for colorectal cancer (CRC) and explores potential future directions [8]. PDT is a minimally invasive, targeted treatment with few side effects that uses photosensitisers and specific light wavelengths to induce cell death in targeted tumour tissues, making it a valuable option for improving CRC treatment outcomes Significant progress has been made in understanding the underlying mechanisms and improving the efficacy of PDT for CRC. The review highlights key advances in PDT techniques, including novel photosensitisers, light sources, and delivery methods. It also discusses ongoing research efforts and potential future directions, such as combination therapies and nanotechnology-based approaches. 

The next four reviews deal with diverse inflammatory conditions. Axial spondyloarthritis (axial-SpA) is a multifactorial disease affecting the sacroiliac joints and spine, leading to inflammation, bone reabsorption, and ankylosis, and its pathogenesis is not yet fully understood [9]. The disease pathogenesis involves genetic, immunological, mechanical, and environmental factors, with HLA-B27 being a significant genetic factor. The innate immune system plays a crucial role in axial-SpA, and the abnormal activity of innate immune cells contributes to disease onset. Additionally, T-cell adaptive responses are involved, leading to the production of proinflammatory molecules. This review explores the molecular mechanisms behind axial-SpA to identify potential therapeutic targets for treatment. 

Asthma is a chronic inflammatory lung disease that displays diverse clinical features and phenotypes with varying phenotypes and affects up to 18% of the population of all age groups [10]. A subset of patients suffers from uncontrolled severe asthma (SA) and does not respond well to standard treatments. Biologic therapies have emerged as promising options, targeting specific molecules involved in the disease. To better understand this heterogeneity, asthma endotyping categorises the condition into different subgroups based on underlying immune responses. Type 2 asthma, driven by T helper 2 (Th2) cells, and non-Type 2 asthma, associated with Th1 and/or Th17 cells, are among the identified endotypes. Thymic stromal lymphopoietin (TSLP), an epithelial-derived cytokine critical in allergic diseases, including asthma, has attracted attention as a therapeutic target, and Tezepelumab, an antibody targeting TSLP, has been approved by the FDA for SA. The review discusses the concept of asthma endotypes and TSLP’s role in SA disease management.

The “Gut-Liver Axis” refers to the interaction between the gut and its microbiota and the liver. This plays a vital role in maintaining health through immune tolerance [11]. It involves bidirectional communication between the gut, microbiota, and the liver, and immune tolerance usually prevents adverse interactions between the two systems. The gut microbiota is essential for digestion, nutrient absorption, and immune response stimulation. However, changes in gut bacterial balance or intestinal barrier function can activate innate immune responses in cholangiocytes (cells lining bile ducts) and result in liver inflammation, leading to liver fibrosis. Understanding the role of essential metals and exploring metal-based treatments could offer potential therapeutic options for cholangiopathies. 

The final review discussing inflammation looks at the potential role of EVs in periodontitis, a chronic infectious disease that damages periodontal tissues, leading to inflammation and bone loss [12]. Current treatment aims to control inflammation, but achieving full tissue regeneration remains challenging. EVs derived from stem and immune cells have shown promise in promoting periodontal regeneration. These EVs contain bioactive materials for cell communication and have advantages over cell therapy, making them a potential alternative for tissue regeneration. Additionally, studies have revealed the role of bacterial and plant-derived EVs in periodontal homeostasis and regeneration. The authors consider the potential therapeutic benefits of these vesicles in periodontal regeneration and explore the challenges and prospects of EV-based treatments. 

A nicely speculative review emphasises the importance of phospholipids in cell membranes with critical functions and their potential as therapeutic targets for cardiovascular diseases, particularly acute myocardial infarction [13]. Given the significant impact of myocardial infarction on the heart, it is crucial to minimise structural and functional changes that lead to heart failure. Phospholipids play essential roles in post-infarction healing, influencing inflammation, proliferation, angiogenesis, and fibrosis. These compounds have been used therapeutically for centuries, but their intricate regulatory functions are only starting to be understood. Moreover, their minimal side effects make them ideal therapeutic agents. Understanding the mechanisms of phospholipids’ action could pave the way for novel therapeutic strategies, benefiting patients with acute myocardial infarction and improving societal and economic outcomes. 

The final three reviews deal with more esoteric and rather important topics. The first looks at the role of metals in disorders of consciousness (DoC) [14]. These result from dysfunction in the cerebral networks governing wakefulness and awareness. Traumatic brain injury (TBI) is a common cause of DoC, affecting brainstem and consciousness pathways. Iron, zinc, and copper play essential roles in the neurophysiology of these pathways. TBI alters neuronal mechanisms involving these metals, leading to neurodegeneration, apoptosis, oxidative stress, and inflammation. Treatments like amantadine, zolpidem, and transcranial direct current stimulation (tDCS) show effects on essential metal-related pathways, and zinc may hold promise as a new therapeutic approach. Understanding metal roles in DoC could pave the way for metal-based drugs as potential treatments. 

The last review is a meta-analysis that deals with mindfulness-based interventions (MBIs) [15]. These have shown positive effects on biomarkers of inflammation and stress in patients with psychiatric disorders and physical illnesses. However, their impacts on subclinical populations are less clear. The authors investigated the effects of MBIs on biomarkers in psychiatric and subclinical populations. MBIs have shown promise in improving mental health and physical conditions, and they may represent an effective treatment option for various psychiatric symptoms and disorders, as results suggest that MBIs may ameliorate biomarker levels in both groups to a small extent. However, low study quality and publication bias may have influenced the results, highlighting the need for larger-scale and well-designed studies in this area of research. 

The final review analyses the role of age and sex in the fatal outcome of COVID-19 [16] by investigating the PUBMED database to gather relevant studies and reviews on mortality due to sex differences and centenarian mortality. It addresses three controversial questions: (1) Have women been more resilient than men? (2) Was the mortality of centenarians lower than that of younger old people? (3) Were older centenarians more resistant to SARS-CoV-2 than younger centenarians? The literature review shows that women are more resilient, but conflicting data exist for centenarian men. Overall, centenarians did not display a lower mortality than less old people. However, in the first pandemic wave of 2020, centenarians >101 years old were more resilient to COVID-19 than younger centenarians. The review discusses factors influencing COVID-19 severity, such as lifestyle, environment, genetics, viral variants, age, sex/gender, and comorbidities. 

The combination of molecular pathology, diagnostics, and therapeutics marks a paradigm shift in medical practice. As precision medicine continues to gain momentum, it has the potential to revolutionise patient care, offering tailored treatments and improved outcomes for individuals grappling with diverse diseases. The integration of these three fields holds the promise of not only enhancing the accuracy and efficacy of disease management but also paving the way for novel discoveries and therapeutic approaches in the pursuit of healthier lives. As technology continues to advance and our understanding of molecular mechanisms deepens, the future outlook for precision medicine is undoubtedly one of the immense possibilities.

Finally, artificial intelligence (AI) is becoming a critical partner. AI-powered algorithms will analyse genomic, proteomic, transcriptomic and metabolomic data and identify disease biomarkers and molecular signatures. They will predict disease progression, therapeutic responses, and potential adverse reactions, leading to more accurate diagnoses and treatment plans. They are likely to be able to detect and quantify subtle molecular alterations for early disease detection and better patient outcomes. Crucially, AI will also accelerate therapeutic development by identifying drug targets, predicting responses, and even designing novel agents, thus facilitating targeted therapies based on individual patient profiles. As AI technology evolves, its integration into molecular medicine will drive significant improvements, shaping the future of healthcare for precision and personalised care.
